# Insanity and Asylums for the Insane—Five Lectures

**Published:** 1841-12

**Authors:** 


### REVIEWS.

---

Art. III.—*Insanity and Asylums for the Insane—Five Lectures.* By W. A. F. Browne, Surgeon and Superintendent of the Montrose Asylum: Edinburgh, 1837.

*Two Reports of the Ohio Lunatic Asylum*, 1839–40.
*Eight Reports of the Massachusetts Lunatic Asylum*, 1835–40.
*Sixteen Reports of the Kentucky Lunatic Asylum*, 1824–40.
*Seven Reports of McLeon Asylum for the Insane*, 1834–40.

The first of these works consists of the substance of five lectures, delivered before the Managers of the Montrose Royal Lunatic Asylum, and answers these five questions:

What is Insanity?
What are the statistics of Insanity?
What Asylums were?
What Asylums are?
What Asylums ought to be?

This is a most useful and interesting book. It is intended both for the profession and for the people at large. No physician can read it without profit; nor other man without satisfaction. It is not so elaborate as Pritchard's work upon insanity; nor so diffuse as that of Esquiral, but it is the result of Dr. Browne's own observation. It is a clear description of what he has seen and felt, and the sufferings of the wretched lunatics of former time, are so faithfully depicted that our sympathies are involuntarily enlisted in their behalf. This treatise does credit both to the head and to the heart of the author: and we would gladly transcribe it entire to our pages, if we had room; as it is, we must be content to give the substance of its most important portions.

In no ailment, which humanity is heir to, has there been so great a change, as to its pathology and its treatment, as in insanity. In ancient time lunatics were considered as possessed of devils, and under the especial influence of evil spirits. It was supposed that these demons acknowledged no law; that they came not in the usual way of human disorder; nor would they yield to the usual remedies. This supernatural disease required a similar power to heal it. These then looked for the especial intervention of Heaven, to arrest the mental vagaries, and then miracles were wrought and the insane restored. But when the age of miracles had passed by, superstition still preserved the same pathology, while religion ceased to offer the same means of relief; and as this pathology was out of the reach of human investigation—so no science bethought itself, to apply any human aid for restoration; and the poor lunatic was left in hopeless alienation; a prey to his wild fancies or his terrible oppression. Unfortunately this theory of the disease has remained till within the last century; and, notwithstanding the immense improvements that were made in the treatment of all other derangements, almost none has been made in those of the mind.

So often were cases of insanity decisively of a moral cast, and connected with fury, madness, and crime, that it was no difficult matter for the world to suppose that these were deserted of Heaven, and given over to the evil one; and that, therefore, the sane were called upon to defend themselves against these dangerous men, and to put such restraints upon them by prisons and chains as would save themselves harmless. Then these outcasts of timid society, were left to pine away in miserable confinement, with no eye to discern the real nature of their disease; no medicine to relieve the nervous system of its incubus; no soothing to calm its distressing excitement.

If the insanity were merely intellectual, and the lunatics only thought wrong; if their passions were still, and their hands harmless, they were suffered to wander as a sort of privileged vagabonds, or live among their friends as a kind of domestic animals. But if they were furious or mischievous, their insanity was a crime, and the penalty corresponding was required of them.

For many ages convents were the principal receptacles of this class of men. In these establishments were strong rooms with solid walls, oftentimes under ground, and as if these were not enough to defend fearful society, chains and handcuffs were ready to be used. Here they underwent the privations which the monks inflicted upon themselves, whether for discipline or for cure is not known. In one convent, in the South of France, ten lashes a day were administered to every lunatic as his share of the monkish benevolence.* To these ascetics, who courted torture and self-chastening, this probably seemed good treatment, and they were almost the only guardians of the insane before the French Revolution.

It was one stage of improvement when hospitals were opened for the insane. These purported to be asylums for the weak and disordered; but they were rather asylums for men's fears, and prisons for the lunatics. They were strongly built with thick walls, narrow windows and barred doors. They were furnished with handcuffs, fetters, muffs, chains, strait waistcoats, confining chairs, and all the means to prevent the maniac from escaping or doing harm to his keepers. "At Rome, iron rings, armed with chains, and fixed in the wall, serve to confine the furious and turbulent maniacs, who are fastened by their necks and feet."† "In one room were two rings fixed to the wall, one ring was to embrace the neck, the other the ankle, and the poor maniac was doomed to stand or suspend himself by the neck."‡ "The accommodations in the asylum at Limerick, appear to be such as we should not appropriate for our dog kennels."|| "One victim was confined

*Browne, p. 101. †Pritchard, p. 249. ‡Browne, p. 118. ||Browne, p. 104.

in one of the oblong troughs, chained down; he had evidently not been in open air for a considerable time, for when I made them bring him out, he could not endure the light. Upon asking him how often he had been allowed to get out of the trough, he said 'perhaps once a week, and sometimes not for a fortnight.' He was not in the least violent; he was perfectly calm."* Esquirol says of the insane in France, "I have seen them naked or covered with rags, with nothing but a layer of straw to protect them from the cold dampness of the ground upon which they lay. They were kept upon food of the coarsest kind; they were deprived of fresh air to breathe, and of water to quench their thirst; and even of the most necessary things of life. I have seen them given up to the brutal supervision of jailors. I have seen them in their narrow cells, filthy and unwholesome, without air or light, chained in such dens as one might fear to confine ferocious beasts."† Similar to these were the abodes of the insane throughout Europe.

The whole arrangement and apparatus of these establishments were for security; to save the public from harm; to defend the keepers and to make them as little trouble as possible; often the manacles were used as the cheapest way of governing them, and thus the expense of attendants was reduced.‡

The keepers of these asylums were corresponding to these prison houses. They were strong and coarse men, with stout muscles and iron hearts, and without fear. Their vocation was to keep and not to cure their subjects. They had neither humanity to pity, nor gentleness to soothe their diseased excitement, nor skill to ascertain or remove its cause. "They knew no other means but fear, to maintain order among them. These officials, who were as barbarous as they were ignorant, knew no other methods to persuade, but the use of chains, whips, and dungeons,"|| and sometimes the mad passions of these keepers, excited by the perversity of their words, reacted upon them, and made the furious ferocity of

---

*Parliamentary Report, 1815. †Des. Maladies, Montales, Iom., 11 p. 400. ‡Parl. Rep. 1815. ||Riel, quoted by Esquirel.

the insane to rage still more violently, and perpetuated the very evil they ought to have relieved. To the uncontrolled passions of such men were the defenceless insane too often given up, and few cared to inquire into the manner in which they administered their trust.

In these hospitals nothing was done to heal the malady of the mind. The old notion of its supernatural origin had, at least, the effect to confirm and perpetuate it, by withholding from the guardians all thought of relief. Nevertheless some singularly ludicrous practices were discovered in a few of the British Hospitals. "In one it was the established rule that every patient should be bled every June, and that each patient should take four emetics a year." "When the physician paid his regular visits, which were few and far between, the patients were arranged in two rows, between which he passed rapidly, receiving reports of their cases at second-hand from the apothecary, and prescribing, guided by some intuitive knowledge, in this fashion—No. 1, a purge; No. 2, an emetic; No. 10, bleeding, &c."*

Of moral treatment there was none for the good of the patient, nor was there power in the hearts or understandings of the keepers and attendants to give it. Their management had the effect to aggravate, not to diminish the disease. No attempt was made to win the confidence of the lunatic, when he entered these sad abodes of alienation. Nor was there labor or amusement prepared to occupy his attention and give him self-control during his sojourn within them. Galled by the needless restraints of confinement and manacles, goaded by the harshness of tyrannical treatment, it is not surprising that the spirit raved in maddened fury, until exhausted, it sunk into hopeless imbecility. As late as 1837, we saw in the Blockley Hospital, near Philadelphia, a poor female confined in a restraining chair made of plank, one strap confined each arm, another the waist, and another passed over the thighs and held her down to her narrow prison. This girl was in a state of furious excitement; she was using the great-

*Brown, p. 122.

est struggles to extricate herself; she was kicking up her feet, endeavoring to strike any one near her; she was boisterous, and spat on any one within reach; she was the very image of a raging fury: and we were told that she had been in this excitement for three years, and the same means of straps and chairs had been as long used to calm her! We trust a similar instance cannot be found elsewhere in America.

These hospitals were once the last resting places of the living maniacs. There they were soon forgotten by their friends without, and too often neglected and abused by their masters within. Very few of the sane world ever entered those abodes of madness and wretchedness to learn their condition, and still fewer of these crazed outcasts returned from that bourne to tell the tale of their sorrows.

Pinel first called the attention of the world to the state of the Lunatic Asylums in France. He had the unprecedented courage in 1792, to unchain their miserable occupants, and to treat them as human beings. Some of them had been chained forty years; some had so long been confined in one crooked position, that their muscles were contracted and their limbs drawn up. Some could not stand; others could not walk: one for almost half a century had not breathed the fresh air, nor seen the vault of heaven. Pinel was thought himself almost a foolhardy monomaniac, to dare to let loose those madhouse prisoners, and men feared for his and for their own safety. But the result was calm and most gratifying. These maniacs were grateful, quiet and peaceable. The story of this deliverance is thrillingly told in almost every work on insanity. It opened men's eyes and the prison doors of the insane: and since that time benevolence and true science have begun to reign over the asylums in France.

About the year 1815, the British Parliament caused investigations into the condition of the madhouses of England, and some frightful revelations were made in consequence. Some of them are stated in the foregoing pages. They are found in the second lecture of Browne; they astonished the nation. A reformation was demanded and begun. "From a blind and hard hearted policy, which sacrificed every tie of justice, and charity, and human fellowship, a sudden transition was made to a system, based upon a knowledge of the human mind and on the common sympathies of our nature, and had for its object the eradication or amelioration of the evil. From darkness they passed into light; from savage ferocity to christian benevolence."* Insanity is now known to be a physical disease, and as amenable to human remedies as any other. It is now an object to restore the lunatic to life and society, not to bury him from sight and hope. And the tender charities, and most faithful skill have taken the place of cowardice and cruelty, in the administration of asylums.

This cheering and honorable change is rather going on now than finished. There is yet much to be done. Greater improvement has been made in some of our American Hospitals than in any of the world; yet others hang far behind the light of the age; their narrow cells, their strong rooms, and their strait waistcoats, have not all been replaced with gentler means of government. But notwithstanding these relics of the former age of ignorance and malpractice, greater progress has been made in the arrangements of the houses for the insane, and in the treatment of their malady, within the last twenty years, than was made in all preceding time. Now these houses are open, airy, and cheerful — formerly, they were strong, close and gloomy. What these American asylums are, we will show from their own documents.

*The Massachusetts State Lunatic Hospital* was built by the State, at Worcester, and opened, January, 1833, for the accommodation of lunatics and persons furiously mad, who were dangerous to be at large; and were therefore confined in jails; and houses of correction for those, who were supported at the expense of the towns, and for others, who were with their friends.

This establishment consists of a centre building 76 feet long

*Browne, p. 129.

and 40 feet wide, and 4 stories high; two front wings each 90 feet long, 36 feet wide and three stories high; and two rear wings each 100 feet long, 34 feet wide and three stories high, extending backwards at right angles with the front. The centre building projects 22 feet in advance of the wings; the front part of this contains rooms for the superintendent and family, and the offices of the physicians and apothecary. In the back part are the dining rooms of the insane.

Half of the width of the front wings is attached to the centre building, and half falls in the rear. "This arrangement allows the long halls of the wings to open into the external air for ventilation." All the wings are divided in each story by a long hall, 12 feet wide in the front wings, and 10 feet wide in the others, running the entire length, and opening at each end outwardly, and allowing a free draught of air through the whole. On each side of each of these halls are the apartments for the insane; these are 10 feet long and 8 feet wide; in each room is a large window with an entire cast iron sash; the upper half of which is glazed, the lower half is open, and a lower wooden sash, glazed, covers, and precisely corresponds with the open part of the iron sash. Every room, for the patients, is supplied with a bedstead—a good straw bed and hair mattress, with sufficiency of blankets, sheets, pillows, &c., and those occupied by the quiet patients have chairs and tables. There are 12 of these halls, and 273 rooms for the accommodation of 238 patients, the superintendent and family, and about fifty overseers, attendants, servants, &c., necessary to conduct its various departments of business and care.

There are, beside these, a chapel, a carpenter shop, shoe shop, wood-yard, farm, and flower-gardens, for the employment of the patients; six large covered verandahs, each 34 feet square, for exercise in stormy weather; carriages and horses for riding; a library; chess, gammon, and chequer boards; musical instruments; and also gravelled walks in the woods, and in beautifully arranged gardens, for the amuse- and occupation of the insane.*

*Reports.

Dr. Samuel B. Woodward was most happily selected for the superintendence of this great establishment, and his eight years of successful management have fully justified the highest hopes of science and humanity. "Every thing," say the trustees, "has been done by the intelligence, benevolence, and firmness of the master-mind of that extraordinary man, who superintends and sways, with consummate skill, the discordant elements, over which he presides, and who has raised the reputation of the State Lunatic Hospital to the rank of a model institution; alike admirable for its humanity economy, and success."* This institution was opened for the worst class of lunatics, criminals, dangerous men, and paupers; the denizens of jails, work houses, poor-houses, and cages, or vagabond wanderers. Some had been imprisoned for various periods, from one to forty-five years. In course of the first year, 107 were received, "who had been adjudged by the courts to be so furiously mad, as to be too dangerous to the peace and safety of the community to be at large." One hundred considered mankind as their enemies, and were, therefore, ready to do violence to any keeper or attendant. Forty had stripped themselves and would not be clothed, even in the severest weather of winter; and many had committed homicide.

One hundred and fifty-three were admitted during the year 1833; of these 105 had been insane more than one year; 20, on an average, three years; 71 from five to forty years; and 14 had no record or friends to tell how long they had been deprived of their reason. A more hopeless class of patients were never gathered together into one asylum. The cost to the State and the towns, for maintaining and guarding these, had been about $40,000 a year.

---

*Eighth report.

*The following table exhibits the statistics of the receptions for eight years.*

| | Old cases. | Recent cases. | Whole number. | Average population of Asylum. | Expense of the Asylum. |
|---|---|---|---|---|---|
| 1833 | 105 | 48 | 153 | 107 | 12,196 |
| 1834 | 55 | 64 | 119 | 117 | 16,941 |
| 1835 | 58 | 55 | 113 | 120 | 16,576 |
| 1836 | 65 | 60 | 125 | 127 | 23,272 |
| 1837 | 95 | 73 | 168 | 163 | 26,027 |
| 1838 | 95 | 82 | 177 | 211 | 28,739 |
| 1839 | 95 | 84 | 179 | 223 | 29,474 |
| 1840 | 87 | 75 | 162 | 229 | 27,844 |
| | 655 | 541 | 1196 | | 180,969 |

*Causes of Insanity.*

| | | | |
|---|---|---|---|
| Intemperance - - | 185 | Abuse of snuff and tobacco | 7 |
| Ill health - - | 185 | Hereditary or with insane kindred - | 361 |
| Masturbation - - | 103 | Periodical - - | 239 |
| Domestic affliction - | 129 | Homicidal - - | 18 |
| Religious - - | 84 | Actual homicides - | 13 |
| Loss of property and fear of poverty - - | 71 | Suicidal - - - | 134 |
| Disappointed affection | 53 | Actual suicides - | 5 |
| Disappointed ambition | 27 | Arising from moral causes - - | 344 |
| Epilepsy - - - | 35 | Arising from physical causes - - | 319 |
| Puerperal - - | 30 | | |
| Injuries of the head - | 16 | | |

Such were the means with which they were to work, and such were the materials on which they were to operate. What was the result of their labours is now to be shown.

It is worth while to observe, that for the State there were two objects, economy and security; the first table shows how the former was accomplished in reducing the cost of maintenance of the pauper lunatics from an average of $50,000 per year to an average of less than $23,000, beside the interest on the cost of the whole establishment; and the superintendent's salery, which is about $2,000 a year. Public security is of course obtained; for none, except such as are either recovered or so far amended as to be pefectly harmless and peaceable, are ever permitted to go beyond the control of the hospital.

But the gain to the individuals who have suffered the pains and deprivations of insanity, and have now recovered the possession of their moral and mental powers, and are restored to the happiness of home and the responsibilities of society, exceeds all pecuniary calculations.

*Table, showing the admissions, recoveries, per centage, and deaths.*

| | Admitted. | Discharged. | Recovered. | Recovered pr. ct. of all discharged. | Recovered per cent. of admissions. | Improved. | Eloped. | Died. |
|---|---|---|---|---|---|---|---|---|
| 1833—Old, - - | 105 | 20 | 11 | 55 | $10\frac{1}{2}$ | 2 | 1 | 4 |
| " Recent, | 48 | 19 | 14 | $73\frac{1}{2}$ | $29\frac{1}{6}$ | 5 | | |
| 1834—Old, - - | 55 | 49 | 10 | $20\frac{1}{2}$ | $18\frac{1}{5}$ | 16 | | 5 |
| " Recent, | 64 | 66 | 54 | $82\frac{1}{4}$ | $84\frac{3}{8}$ | 6 | 1 | 3 |
| 1835—Old, - - | 58 | 60 | 9 | ~~27~~ | $15\frac{1}{2}$ | 20 | 1 | 4 |
| " Recent, | 55 | 52 | 53 | 80 | $78\frac{2}{11}$ | 4 | | 4 |
| 1836—Old, - - | 65 | 49 | 9 | $18\frac{2}{5}$ | $13\frac{11}{13}$ | 9 | 1 | 5 |
| " Recent, | 60 | 57 | 43 | $84\frac{1}{5}$ | 80 | 6 | | 3 |
| 1837—Old, - - | 95 | 63 | 17 | $25\frac{1}{4}$ | 18 | 20 | | 7 |
| " Recent, | 73 | 58 | 52 | $89\frac{1}{2}$ | $71\frac{1}{4}$ | 4 | | 2 |
| 1838—Old, - - | 95 | 70 | 12 | $15\frac{1}{2}$ | $12\frac{11}{19}$ | 18 | | 12 |
| " Recent, | 82 | 74 | 64 | $86\frac{1}{2}$ | 78 | 6 | | 4 |
| 1839—Old, - - | 95 | 97 | 16 | $16\frac{1}{2}$ | $16\frac{4}{5}$ | 27 | | 17 |
| " Recent, | 84 | 71 | 64 | $90\frac{1}{7}$ | 77 | 2 | | 5 |
| 1840—Old, - - | 75 | 85 | 18 | 21 | 24 | 26 | | 11 |
| " Recent, | 87 | 70 | 64 | $91\frac{1}{2}$ | $73\frac{1}{2}$ | 2 | | 4 |
| 8 years. | 1196 | 960 | 505 | | | 173 | 4 | 90 |

In these eight years, 655 old cases were admitted, and 102 were cured, which is 15.57 per cent. upon all admissions; and 541 recent cases were admitted, of which 403 were cured, making 74.50 per cent. Beside these, there were 28 convalescing at the end of the year, which should be added to the number of cures, and this would make 79.74 per cent. recovered of all recent cases that entered the hospital.

Moreover 173 were discharged, improved; being quiet and inoffensive, though incurable, they were restored to their families. This is not all; those who are not discharged, nor even so much improved as to be safe to be entirely at large, are now made comfortable and have many enjoyments. They were before madly furious, in a state of painful excitement or overwhelming oppression; now they are quiet, and have the ordinary physical, and many of the social comforts of life. Their misery is alleviated, and they are objects of tenderness now, rather than of fear and ridicule as they formerly were. It is wonderful how this has been wrought by gentleness and firmness in the superintendent and his assistants. Some maniacs had been for years confined in solitary and cold dungeons, in a constant state of passionate agitation, too desperate to allow any fire in their cells; refusing to wear any clothing, devouring their food like wild beasts, and so mad that their keepers deemed it an act of heroism to enter their rooms; now they sit at a common table with others, calm and self possessed, with their knives and forks, taking their food in order and sobriety; clad in decent apparel, going to bed in composure; uniting in amusements, or joining in labour with cheerfulness and pleasure.* It is not unusual to see two men ploughing in the field, quiet, and attentive to their work, and performing it well, both insane, both having committed homicide, and had therefore been confined many years in prison, as dangerous to be at large.†

"One female had been exceedingly filthy in her habits, had not worn clothes for two years; had been confined in a filthy cell, for the same time, destitute of every comfort and tearing every thing to pieces that was given her. Now she is dressed cleanly, works some, takes her food and at table, in company with sixteen others, sings very pleasantly, when requested, and is very civil and agreeable a large part of the time."‡

We have not room to multiply instances; we quote these as examples to show how much the incurable cases of insanity may be ameliorated. These reports, without intending to

*Report. †Report. ‡3d report.

give all or even any considerable portion, yet detail to us cases of every class, in which the moral and medical means of the asylum had effected this improvement. There may indeed be no hope of the restoration to soundness of mind or affection, but the bitterness is taken from their cup of anguish, and they are harmless toward others, and often useful in their way.

The rules and regulations are printed in the volume of reports published by the State for distribution. These are very minute and judicious. They are established by the board of trustees, and rigidly enforced, upon all the officers attendants and patients. The superintendent is also the physician of the institution. The whole is under his government. He is required to live in the house: to have no other business, except consultations in the town of Worcester, and these principally in cases of insanity. The assistant physicians, chaplain, steward, matron, four overseers, twenty attendants, and eighteen other assistants, also reside in the establishment; are devoted to the service of the hospital, to the exclusion of every other avocation.

The overseers and attendants are required to be men and women of cultivated minds, and of the strictest morality. They must be calm self-possessed and gentle in their manners, and amiable in their tempers. They must be bold and firm, yet mild and gentle in their deportment toward the patients. They must use neither tobacco nor distilled spirits, either at or away from the asylum, as long as they are connected with it. Neither profanity nor angry or taunting language is ever allowed. The least violation of these rules is cause of dismissal from the institution. By this most rigid caution in the selection of attendants, they obtain such as have power to amuse, and dignity to command the the confidence of the patients, and thereby control their mental and moral wanderings.*

*The liberal reward given, for all service in the hospital, enables the trustees to command the best and most faithful attendance. The superintendent has a salary of $2,000, and the support of his family. The assistant physician has $600 and support. The steward and all subordinate officers and assistants are paid such wages as to command the best of their respective classes.

Labor, as a means of cure and amelioration, has been of signal advantage in this institution. Agriculture and gardening are the principal occupations; but they have also carpenters, cabinet-makers, shoe-makers, and tailors, all profitably and satisfactorily employed; for, "patients, who have been brought up to labor, as soon as their first excitement is over, request employment; it is granted, and considered by them as a great favor." "No convalescent recovers so rapidly or favorably as the laborers."

Amusements also are found to be very useful. The patients have their dancing and their social parties. They ride in the carriages; they walk in the gardens, or in the woods, or go into the village, and visit all objects of interest. They read, write, and play at chess and other games, and on musical instruments. They bowl and pitch quoits. The females sew, knit, embroider, aid in the household duties, and, in the summer, cultivate the flower garden. They join in the social and dancing gatherings. Some occupation or other is perpetually offered them, so varied, that they do not become weary of any one, and so successive, that they have no leisure to let their minds run after their delusions.

They have religious exercises in the chapel, morning and evening and on the Sabbath. From 120 to 150 attend them, and no congregation is more orderly or attentive. They have a power of self-control then, which they do not on other occasions; some, who are almost furious elsewhere, are quiet there. Since the chapel was built in 1837, about seven-eighths of the patients have attended service at the same time, and no instance of disturbance has ever occurred.

In this hospital, no harshness or violence is ever used: not a strait waistcoat has ever been there; muffs are not allowed, and rarely confining chairs. Mittens and wristbands are the only methods of restraint, and even these but seldom, and only when the safety of the patient requires it, never for the protection or the comfort of the attendant.*

*"At this moment, November, 1838, out of 230 patients, but one individual, either man or woman, in our wards has upon his or her person, any restraint whatever." 6th Rep. p. 59.

The greatest attention is paid to the physical health of the patients; and for this purpose medicine is frequently given, and often the insanity is entirely removed by this means. In the excited condition of the brain, during the early stages of the disease, medicines operate like a charm, and remove the irritation, upon which the excitement depends. They compose the agitated state of the nervous system and bring about quiet and repose, and the way is open for mental sanity.*

Dr. Woodward and the trustees have published copious annual reports upon the whole regime and progress of the institution. These reports cover a period of eight years, and about 1200 patients of every variety. Dr. W. has done good service to the profession, by his discoveries in the pathology and treatment of this malady, by his detailed descriptions of the means by which he attains his remarkable success, and by his tables of the statistics of the disease. He has analysed his cases with minute discrimination, classified them according to the cause and manifestation of the derangement, to the age, occupation and temperament of the patients, and shown the influence of all these upon the final result. The sum of all these observations is given in 20 tables, which present an invaluable mass of information and philosophical inference relative to the origin, progress and issue of this disease, such as is found in no other treatise upon the disorders of the mind.† We have no room for farther extracts, but refer our readers to the Reports, which are deposited, by the kindness of Dr. W., in the Library of the Louisville Medical Institute, and in the State Library at Frankfort.

---

*8th Rep.

†Of course the proportion of various causes must differ in different communities. Dr. W. found that, in about 1000 cases, 20 per cent. were caused by intemperance; 20 per cent. by ill health; 14 per cent. by domestic troubles; 9 per cent by religious anxiety—60 per cent. were physical causes, 40 per cent moral causes.

Of the curability, in relation to cause, he proves that cases of insanity caused by ill health, 63 per cent.; by religion, 60 per cent.; domestic trouble, 59 per cent.; intemperance, 50 percent.; and masturbation, 27 per cent. were curable.

*The McLean Asylum* for the insane is situated in Charlestown, Massachusetts, about one mile from Boston, and is a branch of the Massachusets general Hospital, in that city. It was established in the year 1818, partly by State bounty, but principally by the means of private subscriptions, and is under the joint control of trustees, chosen by the State and by the subscribers to its funds. It has a small farm and abundant buildings located on a beautiful promontory, partially surrounded by small arms of the sea. With Boston in its front, Cambridge on its right, Charlestown on its left, and the country in its rear, it is in the midst of the busy scenes of active life, and rural enjoyment; near enough to them to have access to all their objects of interest; yet so far separated as never to be interfered with, or be disturbed by them.

The buildings of this establishment have been enlarged and increased from time to time, as the patients multiplied, till now there are ample accommodations for 130 insane, and the superintendant and steward with their families, and all the subordinate officers and attendants; and the whole cost of land, buildings, furniture, horses, carriages, &c., from beginning to end, has been about $250,000. From its great wealth, its magnificent and convenient architectural arrangements, and the abundant means of amusement and occupation, this may be considered as the best adapted to its noble purpose of all the asylums in our country.

It is under the superintendence of Dr. Luther V. Bell, a man who brings to his work a rare combination of skill and benevolence, of practical wisdom and faithfulness. He is aided by an assistant physician, steward, two supervisors, and a large corps of "attendants and nurses of cultivated minds and elevated moral feelings, who engage in their labors with a spirit of patience and self-denial, whose service is not servile, but who are companions of the unfortunate,"* and are able to secure their love, respect, and confidence. The number of these attendants varies with that of the patients. "We have not asked with how small a number we can get along, but

*Dr. Lee's report, 1835.

how many can be advantageously employed?"* "Generally there is one attendant to every four or five patients, independent of particular cases, where from suicidal propensity or other adequate cause, the whole services of one attendant are devoted to a single person."†

The construction of the asylum is so ample and convenient as to admit a more entire and favorable division of patients, into classes according to the nature and developement of their derangement, than is allowable in any other hospital. They can so distribute their inmates as to make more than a dozen different families of each sex; as wholly separated and removed from each other as can be desired. These families or classes have each their proper sitting rooms, sleeping and dining apartments, bathing rooms, &c., and meet each other only as far as is approved, at prayers, in certain kinds of employments and amusements. And by the proper use of these means, they are enabled to dispense almost entirely with restraining measures, or even rigid confinement; so that the strong rooms are not called into use more than three or four times a year. And they rarely have a patient, who does not sit at table with the others and eat with knife and fork.‡

The treatment of the patients is generally the same as that practised at Worcester; upon the modern principles of generous confidence and tenderness, watchfulness and occupation. This institution was the first that had the enlightened courage to try the experiment of mechanical labor, to prove the safety, expediency, and immense utility of putting sharp tools into the hands of the insane, and not the least accident has occurred although many hundreds have had the use of chissels, hatchets, &c.||

This hospital has abundant "and various facilities for keep-

*Dr. Lee's Report. †Dr. Bell's Report, 1839. ‡Ibid.

||And herein our safety lies: the patients, feeling themselves under no restriction, consider that they are placed upon their honor, and their self-respect being called into action, they would not forfeit the confidence and good opinions of the officers, for any consideration. Give a man constant employment; treat him with uniform kindness and respect, and however insane he may be, very little need be feared from him, either of mischief or violence.—Tyler's Report, 1836.

ing every moment occupied—a farm, a highly cultivated garden, a nursery of fruit and ornamental trees, the sawing, splitting and piling wood, a bowling alley, a billiard table for each sex, chess, cards, draughts, newspapers, drawing and surveying materials, a library, six horses, carriages, musical instruments, and other means of labor and amusement, which particular tastes may dictate."*

Mental and bodily occupation are the main reliance for the tranquillizing the excited mind and agitated feelings. Religious worship, the daily prayers, reading the holy scriptures, and singing are found very efficacious for the same purpose. The patients are allowed to attend divine service on the Sabbath, in the churches of their own choice in the vicinity; for almost every denomination has worship within a short walk of the institution. Thirty usually go out to these churches and no disturbance has yet happened.

Beside the great variety of work and amusement, in the hospital grounds, the patients are encouraged to walk abroad, to the gardens, manufactories, monument, the shipping, colleges, and other objects of interest, that abound within a few miles, and no elopement has happened from this liberty.

The whole administration is paternal, affectionate, and confiding; never violent, forcible, or suspicious; no strait waistcoats, hand-cuffs, chains, nor restraining chairs, are there. Mittens are sometimes used to prevent the violent from tearing their clothes, but not one in a hundred is ever thus confined, and never except with the specific authority of an officer. The leathern muff is also sometimes used to allay the vehemence of suicidal propensity, this is also very rare; confidence is, at first, offered the lunatic, "and he is made to understand, that the extent of his privileges will necessarily be dependant on his ability to comply with the rules and to control himself."†

The physicians and every officer and attendant are required to live at the asylum, and devote their whole time and energy to their work; and the least violation of the rules, the

*Bell's Report, 1839. †Bell's Report.

least neglect of discipline over their own tempers or habits, the least failure of gentleness or firmness toward the patients is good cause of dismission. But they are so well selected and so liberally paid, that this rarely happens.

The great munificence of the endowments of this institution, the experience, talents, and devotion of its governors, have enabled it to procure every thing that humanity can desire, skill can suggest or wealth can purchase, for the cure or comfort of the insane, and made it a most desirable abode for those lunatics whose means permit their transportation and residence there, wherever they may belong. And compared with the very great advantage for the recovery of the curable, and for the comfort of the incurable, the expense of $3 50 per week, for residents of the State, and $4 50 per week for residents of other States, is not great.

This being neither a public nor a charity hospital, none are admitted except such as can pay the above prices; but any one can be taken away, whenever the economy or impatience of friends demands it. Accordingly a great many are taken from the institution when they begin to improve, and before the recovery is established. This will explain the large number discharged as improved, but not cured.

In the reports of this asylum no division is made of old and recent cases, but the general results of all. The following table shows the admissions, discharges and condition of the patients when discharged, since the beginning of the asylum.

| YEARS. | Admitted. | Discharged. | Unfit. | Eloped. | Died. | Not improved. | Improved. | Recovered. | Remaining. |
|---|---|---|---|---|---|---|---|---|---|
| 1818 to 1836, | 1311 | 1239 | 13 | 21 | 117 | 218 | 358 | 512 | 71 |
| 1837 to 1840, | 545 | 490 | 8 | 0 | 43 | 52 | 98 | 290 | 125 |
| | 1856 | 1729 | 21 | 21 | 160 | 270 | 456 | 802 | 196 |

Which shows that, for the first 18 years, 41.32 per cent. of all discharged were cured, and 38.8 per cent. of all admitted. During the last four years 59 per cent. of all discharged, and 53 per cent., of all admitted were recovered. In the year 1838, 100 per cent., of all discharged, were recovered, leaving out of the calculation those which were taken away too early, and those who died. In all the 22 years 1 in 88 eloped; and 1 in 11½ died.

*The Ohio Lunatic Asylum* was built, at Columbus, by the State, for the same general purposes as that at Worcester, Mass., and was opened, for the reception of patients, 30th November, 1838. In its plan and its furnishing, in its internal and external arrangements, it is similar to the Massachusetts Hospital. It is situated about one mile from the city of Columbus, on a farm of 30 acres, and contains 153 rooms for the classification and accommodation of 140 patients, and with every means for their comfort and occupation.

During the year 1839 and 1840, of which we have reports, there were received into this asylum, 258 cases, embracing 170 that were of more than one year's duration, and 88 of shorter continuance. These were gathered mostly from places of confinement, jails, houses of correction, and poor houses. Some had committed homicide; others had committed lesser crimes; many were dangerous to the public peace.

This institution was most happily put under the charge of Dr. William M. Awl, whose successful administration has justified his early promise of good to the insane and to the State. He is aided by another physician and a large body of assistants, of suitable character, to command and to compose the people of their charge—all dwelling in the asylum.

As in the hospitals before described, the government of this is kind and watchful, never severe or violent. Harsh instruments of restraint are not allowed, nor any needless confinement. Labor, amusement, and religious exercises are the great moral means; and suitable medication, the physical means used for the cure of the mental disorders. The officers treat all the patients with tender respect, and the attendants secure their good will by affectionate hospitality and attention to their wants. They offer them every allowable privilege, and encourage them to participate in the enjoyment of every pleasure, which their capacity and condition admit. They ride in the carriage; they attend the dancing, social, and musical parties. They have sports on the green; they walk abroad to the town and to the woods, under restrictions. They read, write, draw, and attend the religious services daily and weekly.*

The result of this excellent management shows the recovery of 85 per cent. of all recent cases discharged; 41.17 per cent. of old cases, and 66.66 per cent. of all cases discharged, in the two years.

Beside the entire restoration of reason and happiness to 80 of these afflicted ones, a vast good has been done to the incurables. Those, who before were a pain to themselves and a torment to their friends and dangerous to the public, are now made mostly quiet, comfortable, and even happy and useful. The sting of their mental death is taken away.†

This asylum accommodates 140 boarders, yet is not half large enough for the State of Ohio, and none are admitted from other States. An institution as useful as this ought to be sufficient to accommodate all the insane of its own State.

*The Connecticut Retreat*, for the insane, at Hartford, has been in operation seventeen years, and had charge of 1068 patients, of whom 601 have been cured, which is 56.3 per cent. of all old and recent, epileptic, idiotic, curable and incurable. The last report makes no division of chronic and recent cases; but, from the previous reports, we learn the facts in the following table:

| | Old Cases. | | | Recent Cases. | | | | | |
|---|---|---|---|---|---|---|---|---|---|
| | Admitted. | Cured. | Per cedt. of cures. | Admitted. | Cured. | Per cent. of cures. | Per cent of cures of all cases. | Deaths. | Eloped. |
| 1824—1839. | 464 | 112 | 24.1 | 537 | 451 | 84 | 56.2 | 60 | 0 |

*Reports. †Ibid.

This has been the most successful institution for the insane in the world. The great proportion of recoveries and small number of deaths are proofs of its good management. The means and methods of cure are similar to those in the best hospitals. The farm, gardens, and joiners' shops, afford abundant and useful labour. The carriage, walking, library, games, pictures, are sources of profitable amusement. Beside these, the women sew, knit, dance, and sing. Religious worship, morning and evening, and on the Sabbath, are now looked forward to by the patients with pleasurable anticipation.*

Dr. Amariah Brigham has been superintendant for one year, and has so far answered the high expectations of the friends of the Retreat. He has, for co-operators, an assistant physician, chaplain, thirteen attendants to take charge of seventy-nine patients, and four others, who have each the care of one patient, and ten other assistants for the household duties.† All reside at the house and are well paid. These are required to treat the patients with kindness and respect. Strait waistcoats, restraining chairs, and all these relics of barbarism find no place there.

This hospital can accommodate 100 patients, and had, in March, 1841, 79. These, if they belong to the State, pay $3 50 per week; if out of the State. $4 per week, and are well compensated for their money, by skilful attention and almost certain restoration to health.

*The Vermont Asylum* was established at Brattleborough, in 1816, partly by private bounty and partly by State grants, on condition that the poor of the State be received at $2 per week; others pay $3 per week. One hundred patients can be accommodated, and eighty-one were there, at the date of the last report, October, 1840. During last year 73 were admitted; 142 enjoyed the benefit of the institution; of these 54 were discharged, 1 eloped and 6 died. Of the old cases discharged 28 1-5 per cent. were cured. Of the recent cases 88 1-5 per cent., and of all 54 per cent. had recovered.‡

*Dr. Brigham's Report. †Ibid. ‡Dr. Rockwell's Report.

Dr. Wm. H. Rockwell has the whole charge, and manages it according to the plan, and with the success, of Dr. Woodward, at Worcester. A farm, shops, riding, reading and religiuos service are used among the moral means of cure.

*The Bloomingdale Asylum*, near New York city, is one of the finest institutions in the United States. It has beautiful grounds, means for riding, chapel for religious exercises, and a library, and accommodates about 140 patients. It has been 20 years in operation, and received 2486 patients, and cured 77 per cent. of the recent cases, and 11 per cent. of the old cases, and $45\frac{7}{8}$ per cent. of all. Two hundred and twenty-two have died, which is $8\frac{6}{7}$ per cent. of the whole. At the date of the last report, 131 were in the asylum under the superintendence of Dr. Wm. Wilson, aided by 20 assistants and other subordinates.*

The particular method of treatment is not described in the report before us; but, whatever it may be, such success as the tables show, argues a proper administration.

*The Friends Asylum*, at Frankford, near Philadelphia, was opened in 1817, and is now under the management of Dr. Pliny Earle, the resident physician. Six hundred and twelve patients have been admitted; and 259 were cured, 143 improved and 84 died This gives 42.3 per cent. recovered, and 13.7 per cent. died, of all that were in the asylum in 24 years.

This is an admirable establishment, under the control of the Society of Friends. It is large, elegant, well arranged, and capable of accommodating 65 patients. There is a farm, a grove, a little deer park, carpenters' shop, basket-makers' shop, library, cabinet, museum of natural history, a circular railroad, and a carriage and horses for the use of the inmates. Labour and amusement, religious worship, and lectures on science are very efficacious there. "Gentle manners, kindness, and the greatest mildness form the ground work of the system, by which the feelings of the patients are generally controlled and interested."† "There are no means resorted to, no system of

---

*Report for 1840. †Journal Med. Science.

treatment pursued of which every physician would not avail himself in his private practice."*

*The Pennsylvania Hospital for the insane* is situated about two miles westward of Philadelphia. It is a splendid establishment, and no expense has been spared in its erection. When finished it will accommodate 200 patients. There is attached to it, a farm of 42 acres, and all is under the care of Dr. Thomas S. Kirkbride, whose character promises much for the success of the institution. The insane, formerly in the Pennsylvania Hospital in Philadelphia, are removed to this new building.†

*The Maryland Hospital*, near Baltimore, can accommodate 150 patients. In six years it has received 393 patients, and cured 135, which is 33 per cent.; 34 have died which is 8 per cent. The means of cure are labor, agricultural and mechancal, gardening, and carpentering, amusements of walking, riding, fishing, reading, and games, and, not least important, religious worship. This is under the care of Dr. Wm. Fisher.

In Virginia there are two asylums, one at Williamsburgh, the oldest institution for paupers in the Union. It contains 60 patients: We have no report from it.

The *Western Lunatic Asylum* is at Staunton, Virginia, a State establishment, under the charge of Dr. Francis T. Stribbling. It has a farm, library, horses, carriages—a piano and other instruments of music. They have labour on the land, and cutting wood, parties, balls, games, sewing, knitting, and religious exercises. From 1828 to Nov. 1839, there were 157 patients admitted and 47 cured; of the recent cases 83 per cent. recovered.

The *Maine Asylum*, at Augusta, has been in operation only two months at the date of the last report. It had then received thirty patients, and had room for ninety more, and

---

*Report, 1841.

†Earle. Visit to thirteen asylums, for the insane, in Europe; and notice of other institutions in Europe and America. This is the most comprehensive mass of statistics on this subject that we have found. It contains a notice of forty British and continental hospitals, and twenty-two in the United States.

the usual means of occupation and amusement that are found in the best hospitals.

There is an asylum in Columbia, South Carolina, built by the State at the cost of $100,000, and one in Milledgeville, Georgia. A new building has been erected in the yard of the Charity Hospital, in New Orleans. But so narrow are the external accommodations that little can be promised for the lunatic, except shelter and protection. A large hospital is established by the State of Tennessee, in Nashville, for poor lunatics. There are city asylums in Boston, New York, Philadelphia, Baltimore, and Washington for the insane poor.

The *Kentucky Lunatic Asylum* is beautifully situated on the confines of the city of Lexington, in the midst of a small farm, on a gentle declivity. The hospital consists of a centre building 66 feet long, two front wings, each 62½ feet long, and two lateral wings extending backwards from the outer end of the front wings, and 22 feet wide; making the entire front of the building 235 feet in length. These afford room for 130 patients. There are 18 acres of land for exercise, but no shops nor other means of occupation or amusement. There is one other building for the incurables; and also one 20 feet square and two stories high, containing 16 rooms, for the violent and unmanageable. A part of the whole is surrounded with a high fence to prevent escape. Originally there were provided hand-cuffs, strait-waistcoats, and other strong apparatus for confining and restraining the patients according to the notions of the time, for the treatment of this class. This institution was primarily intended for lunatics who were mad and dangerous to be at large, and for insane paupers; but, afterwards, others were admitted from this and other States, by payment of $2 50 per week.

A little land is cultivated, vegetables for the family are raised, in the garden, by the labor of the patients. The nursing the sick, the care of the rooms, the cooking, the household work in general, and the making the clothing for the pauper insane, are principally done by the inmates. Yet the report for 1840, out of 201 patients, gives only 12 as at work; and of all those, who had entered within the last ten years, only 4 are recorded as at work. If there be no mistake in this record, and those of 1839 and 1838 are very similar, we must conclude that labor is not required of the recent cases; nor is it used as a constant remedial means.

This asylum is under the supervision of five commissioners, who make annual reports, to the Legislature, of the condition and the yearly history of their charge. We have these reports, for the last seventeen years, now before us; and have studied them, with great care in the vain hope of ascertaining precisely how far, and in what manner the whole object of the institution had been accomplished. The substance of these is comprised in a table of the patients. This, in several columns, gives us the date of entrance, the age, sex, county or State, and present condition of the lunatic, and whether married or single. There is one singular column, which purports to give the diseases, but which is a strange mixture of causes and effects. Here we find under the same head, as insane disease, idiocy, catamenia, mania, blow on the head, melancholia, puerperal, hard study, epilepsy, lunacy, dolore, grief, á potu, hysterics. This column leaves a double question still open: we wish to know how the insanity originated, and what is its present form. Every one, conversant with this subject, knows that the puerperal state, epilepsy, intemperance, hard study, the moral affections and physical injuries are causes, and may produce any of the forms of insanity; and that mania, and melancholy, and idiocy, are effects, and may be brought on by either of the foregoing. Idiocy may be congenital, and in this sense it is used by most writers. In these reports it includes both those who were born idiots, and those who are in the last stage of dementia,* the fourth degree of inappetency of Pritchard: for we find, on comparison of reports of various years, that the disease of some is at first called mania or epilepsy, or á potu, and afterwards idiocy.

These reports give us no account of the length of the dis-

*This mistake is not peculiar to Kentucky, for Esquirol says, of some French writers, "They have confounded idiots with demented persons, and the reverse, and often with monomaniacs." Tom. 11, p. 283.

ease, previous to entrance into the asylum; nor of the resources for occupation of the patients, nor of the principles or details of medical practice; nor of the condition of those who were discharged previous to 1839. So far then as to giving the world any knowledge of the causes and nature of insanity, in Kentucky, or of its duration and curability, or of the internal economy and management of our State asylum, or its means for curing its lunatic residents, these annual reports disappoint our hopes. Probably they were prepared, not by any medical officer, but by the steward or commissioners or other persons, who are not supposed to be acquainted with the minutiæ of this disease.

Governor Adair, in his message to the General Assembly in 1821, urged as one reason for the establishment of this asylum, that it "would prove highly beneficial to the medical school, which would, in time repay the obligation by useful discoveries in the treatment of mental maladies."* This was the early hope; but so far from making any such discoveries, the administration of this hospital has not even adopted and practiced upon the discoveries, which have been made and published by others. Nor have any contributions been made to science from that source, excepting two valuable articles upon the legal and statistical history of the institution, from the pen of Dr. Theobald, in the Transylvania Journal, in 1829 and 1830.

Nor ought this to have been expected. The treatment of insanity is a specific study. It requires one's whole and undivided attention. Yet no physician has ever been so employed by the State, for this asylum, as to be able to give it his whole time and thoughts. The most that is done, is to hire one to visit the establishment daily, and this for a very small remuneration.† Living at a distance from the institution, with the necessary cares of miscellaneous practice, and the necessity of obtaining support by this, the most that

---

*Journal of Senate of Keutucky, 1821, p. 19.

†Cost of medicine and medical attendance for 1838, $341 88; for 1839, $373 38; for 1840, $264 04, being $970 30 for three years medical attendance and supply of medicine for 327 insane patients, and a constant average of 125. Reports.

could be expected of him, is that he should attend to the incidental diseases of the insane, but not to give that constant, anxious, and unremitting attention, which is needed for the cure of mental derangement.

The services of the medical faculty of Transylvania University were gratuitous and occasional, and therefore irresponsible for the permanent care of the insane. As this practice is entirely out of their usual routine of professional business and study, this generous offer of consultation aid, must have been of much less value than in ordinary diseases; for the incidental and temporary counsel, of all the most gifted of the profession, will avail little, in the cure of insanity, compared with the unremitting watchfulness of a single physician, exclusively devoted to it.

From May, 1824, to January, 1841, the asylum received 841 patients. Of these 337 died, (including 43 who died of the cholera, in 1833,) 78 eloped, 284 were discharged and taken away by friends, and 142 remained. We have no means of ascertaining what was the condition of those, who were discharged or taken away previous to 1839, whether they were cured, improved, or stationary. Nor what proportion of these were discharged, by request of friends, before the hospital had exerted all its influence for their restoration, or by desire of the commissioners, after they had ceased to hope. The following table we copy from the reports of 1839 and '40. It gives a concise statistical history of the institution.

I.

*Showing the admissions, discharges, and deaths, in each year from the opening of the Asylum, in* 1824, *to* 1st *January*, 1841, *and the relative proportion the two latter bear to the first.*

| Year. | Admitted. | Discharged | Died. | Remained. | Total in House. | Average number. | Per cent. discharged. | Proportion discharged one in | Per cent. died. | Proportion died, one in |
|---|---|---|---|---|---|---|---|---|---|---|
| 1824, - - | 54 | 13 | 2 | 39 | 54 | 28.00 | 24.07 | 4.15 | 3.07 | 27.00 |
| 1825, - - | 39 | 15 | 7 | 56 | 78 | 46.83 | 19.23 | 5.20 | 8.97 | 11.14 |
| 1826, - - | 33 | 17 | 4 | 68 | 89 | 58.83 | 19.10 | 5.23 | 4.49 | 22.25 |
| 1827, - - | 38 | 22 | 13 | 71 | 106 | 71.50 | 20.75 | 4.81 | 12.26 | 8.15 |
| 1828, - - | 35 | 12 | 9 | 85 | 106 | 77.41 | 11.32 | 8.83 | 8.49 | 11.77 |
| 1829. - - | 41 | 12 | 22 | 92 | 126 | 91.08 | 9.52 | 10.50 | 17.46 | 5.72 |
| 1830, - - | 31 | 19 | 16 | 88 | 123 | 91.08 | 15.44 | 6.47 | 13.00 | 7.68 |
| 1831, - - | 43 | 18 | 11 | 102 | 131 | 93.50 | 13.74 | 7.27 | 8.39 | 11.90 |
| 1832, - - | 42 | 26 | 15 | 103 | 146 | 103.33 | 17,80 | 5.61 | 10.27 | 9.73 |
| 1833, - - | 40 | 14 | 60 | 69 | 143 | 83.00 | 9.79 | 10.21 | 41.96 | *2.38 |
| 1834, - - | 52 | 27 | 10 | 84 | 121 | 76.41 | 22.31 | 4.48 | 8.26 | 12.10 |
| 1835, - - | 54 | 24 | 21 | 93 | 138 | 91.41 | 17.38 | 5.75 | 15.21 | 6.57 |
| 1836, - - | 57 | 24 | 26 | 100 | 150 | 101.08 | 16.00 | 6.25 | 17.33 | 5.76 |
| 1837, - - | 68 | 33 | 22 | 113 | 168 | 106.91 | 19.64 | 5.09 | 13.09 | 7.63 |
| 1838, - - | 63 | 25 | 29 | 122 | 176 | 117.09 | 14.20 | 7.04 | 16.47 | 6.07 |
| 1839, - - | 86 | 37 | 35 | 136 | 208 | 132.49 | 17.97 | 5.62 | 16.83 | 5.94 |
| 1840, - - | 65 | 24 | 35 | 142 | 201 | 136.93 | 11.94 | 8.37 | 17.42 | 5.74 |
| 17 years, | 841 | 362 | 337 | | | 88,64 | 43.10 | 2.32 | 40.65 | 2.46 |

The third column, the discharged, includes all who were cured, improved, and stationary; and were discharged by the commissioners or were taken away by friends, or eloped. But these are more satisfactorily distinguished in the reports of 1839 and '40, which we give, condensed.

II.

*Statement of the manner of discharge and condition of those who left the Asylum in* 1839–40.

| | Patients in the Asylum. | | | Taken away. | Died. | Eloped. | | | | Discharged. | | Total cured. | Total improved. |
|---|---|---|---|---|---|---|---|---|---|---|---|---|---|
| | Previously. | Admited. | Total. | | | Cured. | Improved. | Stationary. | Total. | Cured. | Improved. | | |
| Old cases, - - - - - | 82 | 79 | 161 | 8 | 44 | 7 | 4 | 3 | 14 | 6 | 4 | 13 | 8 |
| Recent cases, - - - | 11 | 50 | 61 | 4 | 8 | 2 | 2 | | 4 | 12 | 6 | 14 | 8 |
| Idiots and epileptics, | 29 | 22 | 51 | | 18 | | | 3 | 3 | | | | |
| Total, | 122 | 151 | 273 | 12 | 70 | 9 | 6 | 6 | 21 | 18 | 10 | 27 | 16 |

*This year 43 died of the Asiatic cholera.

The following table shows the comparative success of one Belgian, three French, thirteen British, and ten American Lunatic Asylums.

III.

*Comparative view of twenty-seven Hospitals.*

| | Time. | In Hospital. | Cured. | Per cent. of cures. | Per cent. of deaths |
|---|---|---|---|---|---|
| *European.* | | | | | |
| Utrecht,* - - - - - | 1832 to 1837 | 255 | 105 | 40.07 | 21.56 |
| Charenton* - - - - | 1826 to 1834 | 1205‖‖ | 516 | 42.82 | 26.64‡‡ |
| Saltpetriere,* - - - | 1801 to 1813 | 3007 | 1625 | 54.04 | 27.02 |
| Esquirol's,* - - - - | | 335 | 173 | 51.64 | |
| *British.* | | | | | |
| Bethlehem,† - - - | 1819 to 1833 | 2445 | 1124 | 45.56 | |
| St. Lukes,* - - - | 1751 to 1801 | 6458 | 2811 | 43.52 | |
| Wakefield,* - - - - | 1819 to 1836 | 2242 | 991 | 44.20 | 31.64 |
| Lancaster,* - - - - | 1817 to 1832 | 1750 | 697 | 39.82 | 24.29‡‡ |
| Stafford,* - - - - - | | 1000 | 429 | 42.90 | |
| Retreat near York,* | 1796 to 1835 | 508 | 236 | 46.43 | |
| York,* - - - - - - - | 1815 to 1837 | 1131 | 387 | 34.21 | |
| Cork,* - - - - - - | 20 years | 1431 | 751 | 52.48 | |
| Gloucester,† - - - | 1823 to 1832 | 516 | 231 | 44.76 | 11.00 |
| Liverpool,‡ - - - - | 1836 | 102 | 46 | 44.65 | 10.00 |
| Glasgow,‡ - - - - | 1836 | 122 | 51 | 41.80 | 10.00 |
| Lincoln,‖ - - - - - | 1820 to 1838 | 715 | 285 | 39.87 | 16.27 |
| Suffolk,§ - - - - - - | 5 years | 362 | 167 | 46.10 | 28.07 |
| *American Asylums.* | | | | | |
| Hartford,¶ - - - - | 1824 to 1840 | 1001 | 563 | 56.02 | 5.9 |
| McLean,¶ - - - - | 1836 to 1840 | 616 | 290 | 47.00 | 6.2 |
| Bloomingdale¶ - - | 1823 to 1840 | 2496 | 1145 | 45.87 | 8.87 |
| Worcester,¶ - - - | 1833 to 1840 | 1196 | 506 | 42.41 | 7.5 |
| Frankford,¶ - - - | 1817 to 1840 | 507 | 214 | 42.21 | 14.00 |
| Vermont,¶ - - - - | 1837 to 1840 | 239 | 98 | 41.00 | 4.6 |
| Ohio,¶ - - - - - - - | 1839 to 1840 | 258 | 80 | 31.00 | 8.5 |
| Staunton,* - - - - | 1828 to 1839 | 157 | 47 | 30.00 | 9. |
| Kentucky,** - - - | 1839 to 1840 | 273 | 27 | 9.89 | 25.6 |
| Hudson,†† - - - - | 1840 | 84 | 22 | 26.02 | 5.7 |

*Earle. †Pritchard. ‡British Medical Almanac. ‖Hill. §Crowther, quoted by Woodward. ¶Reports. **Reports of 1839–40. This includes the accumulated old cases, and idiots and epileptics of former years. ††Boston Medical Journal. ‡‡This per centage of deaths is taken from another table in Earle, and does not cover exactly the same years as the other columns. ‖‖This does not include 274 paralytics, 62 epileptics, and 15 idiots; but it includes 492 incurable cases which had been in the asylum many years previous to 1826.—Esquirol: Tom. II. p. 690-91.

### IV.

*Of the per cent. of all cases in three Asylums*, 1839–40.

| | Patients in the Asylum. | | | Cured. | | Per cent of cures. | | Per cent of deaths. | Eloped. |
|---|---|---|---|---|---|---|---|---|---|
| | Total. | Old cases. | Recent cases. | Old cases. | Recent cases. | Old cases. | Recent cases. | | |
| Massachusetts, | 559 | 372 | 187 | 34 | 128 | 9.14 | 68.45 | 6.5 | |
| Ohio, - - - - - | 258 | 170 | 88 | 21 | 59 | 12.34 | ~~31.38~~ | 8.5 | 1 |
| Kentucky, - - - | 273 | 212 | 61 | 13 | 14 | 6.13 | 22.95 | 25.63 | 21 |

67

### V.

*Of the per centage of all cases discharged*, 1839–40.

| | Discharged. | | | Per cent. recovered. | | | Per cent. died. |
|---|---|---|---|---|---|---|---|
| | Old. | Recent. | All. | Old. | Recent. | All. | |
| Massachusetts, | 182 | 141 | 323 | 18.68 | 91.20 | 50.01 | 11.00 |
| Ohio, - - - - - | 51 | 69 | 120 | 41.17 | 85.50 | 66,66 | 18.00 |
| Kentucky, - - - | 97 | 34 | 131 | 13.04 | 41.17 | 20.06 | 53.00 |

### VI.

*Of the per centage of all admitted*, 1839–40.

| | Admitted. | | | Cured per cent. | | |
|---|---|---|---|---|---|---|
| | Old cases. | Recent. | All. | Old. | Recent. | All. |
| Massachusetts, | 182 | 159 | 341 | 18.68 | 80.05 | 47.00 |
| Ohio, - - - - - | 170 | 88 | 258 | 12.35 | 67.00 | 31.00 |
| Kentucky, - - - | 101 | 50 | 151 | 12.87 | 28.00 | 18.00 |

The third of these tables shows the comparative success of twenty-seven hospitals in curing the insane. The fourth column of figures shows the per cent. of recoveries of all that were in the asylums during the time specified. The last column shows the per cent. of deaths.

The three institutions, compared in the other tables, are all established on the same principles and for the same general purpose—to receive the pauper lunatics and those who were dangerous to be at large. Table No. IV. shows the per cent. of cures upon all, enjoying the benefits of the hospital, for two years. No. V. shows the per cent. upon all discharged, and No. VI. upon all admitted.

The asylums in Kentucky and Massachusetts being old, contain an accumulation of incurable cases, which had resisted the efforts of previous years; therefore table No. IV. would show a per centage of cures in favor of Ohio, all of whose cases had at least been untried. The asylums in Ohio and Massachusetts, on account of their crowded condition, are obliged to discharge those who are incurable, but who are not dangerous to be at liberty; therefore the per centage in the table No. V., reckoned upon the discharges, might show a number of cures in favor of Kentucky. This last asylum undoubtedly admits a much greater proportion of idiots and epileptics (22 in 151)* than either of the others, hence the last table might exhibit a result against it as to the old cases, (for in that class are included all the epileptic and idiotic,) but not as to the recent cases.

Taking these three bases of the calculation, we believe these tables will show a true comparison of the influence of these institutions over their deranged inmates. But whatever may be the method of comparison, we cannot fail to be struck with the great number of deaths, the frequency of elopements, and small number of recoveries in our asylum.

---

* "Very few were brought to the institution, except those of the very worst class of patients." Transylvania Journal, III., 88.

The census of 1840 gives 317 lunatics supported at public charge, and 516 at private charge, in Kentucky. Of these only 176 were in the asylum. The State allows pauper lunatics to be supported at their homes, out of the public treasury, if they be peaceable, and if that cost be no more than it would be in the asylum. Of course then the friends would be apt to send the worst, the most excited, and most fatuitous to Lexington.

In the last two years, with an average of 134,5 in the house, at Lexington, 35 died each year. In 17 years, out of 841 patients admitted, 337 died, which is 26 per cent. on the average annual population; and, after deducting the 43 deaths of cholera, 36 per cent on all admitted. In 22 years, the McLean asylum admitted 1856 patients; of these 160 died, which is 8.6 per cent. And in the Bloomingdale asylum, during the period of 17 years, 8.87 per cent. died. In the French and British asylums, the proportion of deaths is much higher than in the American, excepting that of Kentucky. The deaths in the European vary from 7 per cent. to 48 per cent. depending both upon the character of the patients admitted and the management of the hospital. Insanity is not, in itself, a very dangerous malady; yet it so affects the constitution, as to leave it open to the attacks of other diseases, and with far less power to resist them. Dr. Theobald says,* of the Lexington asylum, "The degree of mortality is to be accounted for, to a considerable extent at least, by reference to the wretched character and condition of a large majority of the cases occurring. A more particular account of these would have presented a number very infirm from advanced life, and others laboring under great bodily debility from other causes." "A disease sometimes in the form of dysentery, but much more frequently of severe diarrhœa, has prevailed in the institution to an unusual extent, and has terminated the existence of a large majority of those who have died." Dr. T. thinks this is owing to the want of regular and sufficient exercise and vicissitudes of temperature, as it occurs mainly in the extremely fatuitous and idiotic patients, and in the spring and fall seasons. Mr. Farr† says, "the mortality of 7 per cent. annually in the asylum at Gloucester, may be fairly ascribed to insanity. The excess above this must be attributed to the diseases generated by the limited space in which the unhappy lunatics are confined, to the collection of large numbers under the same roof; the impurity of the atmosphere; the want of exercise and warmth; the poor unvaried diet, and the deficiency of medical attendance."

*Transylvania Journal, 1830. †London Lancet, May, 1841.

These causes of disease require the greatest watchfulness to guard against them, both in Britain and America, and in the most improved institutions, they are resisted and overcome. How far they operate in conjunction with the causes, before stated by Dr. Theobald, in producing the extraordinary mortality of the Kentucky asylum, is only to be learned by a thorough investigation of that institution, and comparing it in its internal arrangements and administration with other asylums, in which the mortality is much less.

*Elopements.*—Seventy-eight eloped from the asylum at Lexington, out of 841 patients in 17 years, which is about 1 in 11. We have examined the reports of ten other American asylums, which run back variously from one to twenty-four years, and include 5325 patients, and find that of all these only 32 have eloped, which is 1 in 166. Here is an extraordinary and unaccountable difference.

The Lexington asylum has a high fence to prevent the escape of the patients. Whereas most others have no other enclosure than such as surrounds any private residence. But they have a large corps of attendants, who, by their close watchfulness, and by securing the confidence of those under their charge, retain them within their control.

These reports, which we have now reviewed, show that while most of the American asylums are doing more for the cure of insanity than any others in the world, ours is doing the least—that while others are curing from seven to nine-tenths of all that have been deranged one year and less, and from two to four-tenths of those who have been insane a longer time, our asylum has cured no more than one-tenth of old cases and four-tenths of the recent ones. It is plain then that we are not doing in Kentucky, for this unfortunate class, all that the present state of science can accomplish, and that humanity expects of us.

This deficiency is to be partially accounted for, by the character of the patients sent to the Lexington Asylum. For although insanity is primarily as curable in this as in any other State, yet as the most obstinate and incurable cases are selected, out of all in the State, to be sent to the hospital, the chance of recovery must be less, and the danger of death greater than it is in other institutions.

Another cause is in the structure and administration of our asylum. This was established, when insanity was supposed to be mostly without remedy, and it was not thought needful to make provision for its cure. It was built for the protection of the public and for the security of those lunatics who were existing in strong places or strolling over the country. To maintain them comfortably and economically was all that philanthropy asked or medical science promised. Recovery was a secondary object, rather than the main principle. Public security and economy were obtained, for the dangerously mad are confined, and the expense of supporting the whole is much reduced. From 1802 to 1822 the cost to the State of maintaining pauper lunatics had increased from $892 to $15,492 per year. But by the establishment of the asylum, this fearfully rapid augmentation of expense was arrested, and the annual charge was even reduced below that of the five years, from 1819 to 1823. During these last years of the old system of supporting this class of paupers, they cost $23 per 1000 inhabitants; and during the last 12 years of the new system, from 1829 to 1840 they cost the State only $17 per 1000 inhabitants.

The establishment of this hospital was then indeed an improvement upon the old plan of supporting the lunatics, in their respective counties, under the care of committees. They gained in comfort, and some of them were restored to sanity and to their friends. But since 1824, the whole theory and practice respecting insanity has changed. It is now found to be a physical disease, and to be as curable as any other acute disorder; and asylums are now constructed and administered to correspond with the improved notions of the nature of the malady and of its treatment. But while others have been advancing and exhibiting increased success, year by year, our asylum has remained the same.

Is this state of things necessary? Cannot we do as much for the lunatic in Kentucky, as is done in other States? Most surely we can. But first we desire, that the Legislature would appoint a committee to examine the asylum and consider the whole matter. Let them investigate the means and facilities for the treatment of the insane, and the principles and details of their management. Let them compare this with the best asylums and see wherein they differ, and why there is such a wide difference in their good results. Let them read the full and satisfactory reports of Dr. Awl and Dr. Woodward, and see how far all, that is good and useful in their hospitals, can be adopted here. We are aware that committees have gone from Frankfort to Lexington, every winter for this purpose; but their examinations have not been so thorough, nor their reports so full and minute, as we now hope to see. And we doubt not, such a procedure would discover the causes of our deficiencies, and suggest suitable remedies.

Every successful institution has an experienced physician, devoted exclusively to the insane with ample remuneration to induce him to give up every other occupation. Living in the asylum, he is able to give his whole time and energy to studying the character and symptoms of his patients; and to the cure of their disease. Without this advantage, our asylum must, as it has done, fail to reap the success of others.

We want occupation of all sorts, labor and amusement, more agricultural employment, shops for mechanics, exercise abroad by riding and walking, games, books and periodicals in the house. We know that it was thought some years since at Lexington, unsafe to put edged tools into the hands of the insane, lest their propensity to destruction might prove injurious to themselves or others.* But subsequent experience has shown, that they may be intrusted with sharp instruments, and no fears arise for their improper use of them. Hundreds have worked with chissels, hatchets, &c., in the McLean asylum, and not the slightest accident has occurred.† Patients are employed as carpenters in the hospitals of Utrecht and Sonnestein, at Hanwell, Richmond, and Wakefield in Europe, at Worcester, Charlestown, Frankford, and Co-

*Trans. Jour., III, 83. †Bell's Rep., 1839.

lumbus, in this country. "Shoe-makers, tailors and carpenters have been, for years, tried and found to work as diligently as when at liberty. I cannot see or admit any limit to the application of this principle."*

Religious exercises have been elsewhere exceedingly beneficial, and several asylums have a regular chaplain devoted to the moral and religious treatment of the insane and with singularly good effect, to compose and restore them.

A complete examination of this asylum and comparison with others, will suggest other means of reform needful to give it all the facilities for curing the insane, which modern science has developed in other places.

To do all that may be required, to employ a physician exclusively, and such a number of assistants, and of such character as to have the best influence over the patients—to provide shops, horses, carriages, library, agricultural and mechanical tools of various sorts, and all the means of occupation, light and laborious, will, at first, increase the expense to the State. But in the end it will be a saving of money, for by this additional outlay, the recent cases may be mostly cured, and a much greater proportion of the old cases than now recover. It is much cheaper to cure than to support the patients in unrelieved insanity. In the former case, a few months is all that would be needed to support them. In the latter, they must be a tax upon the public as long as they live. The 142 in the asylum at the beginning of this year, had resided there, on an average, forty-three months. While those who were cured, had lived there on an average, only eleven months. So far as this comparison goes, it proves that the curable cost the State only one-fourth as much as the incurable.

We propose, also, that the Assembly repeal the law of 1825, which permits such lunatics or idiots as are quiet and peaceable, and can be maintained at the same cost, at their homes as at the asylums, to remain in their respective coun-

*Browne, p. 96.

ties, under the charge of their committees, and draw the cost of their support from the public treasury.

And that the Legislature should withdraw all out of door allowance for the support of any lunatic away from the asylum, until he or she shall have been placed under its care, long enough to decide whether the case were curable or not. This would compel every pauper to be sent to Lexington as early as possible, after the attack of insanity, and thereby secure to the asylum a much greater proportion of recent cases and diminish the old ones. This would very much lessen the cost of maintaining lunatics. Dr. Woodward demonstrated, that the average cost of maintaining 25 recent cases, both before they entered and during their residence in the hospital, was $56 each. While that of supporting as many of the oldest cases was $1903 each.* Without doubt, similar results would be obtained from comparison of these two classes in this State.

We have seen, that the chance of recovery in every hospital is much greater for the recent than for the chronic cases. Dr. Woodward's tables show, that, 88 per cent. were cured of those insane less than one year; 57 per cent. of those insane from one to two years; 34 per cent. of those insane from two to five years; and 11 per cent. of those insane from five to ten years.* Dr. Tuke, of the York asylum, gives 79 per cent. of cases of less than three months; 44 per cent. of those from three to twelve months; 25 per cent. of those of more than one year's standing, as curable.† Dr. Burrows cures 90 per cent. of very recent cases.‡ Dr. Bell, of the M'Lean asylum, cured 100 per cent. of all that were not interfered with, in 1838.|| This is the greatest success on record—and could not always be expected even in that excellent institution. We see then, that insanity of very short standing is one of the most curable of all acute disorders. We hardly think, that a table of as many cases of dysentery, fever or pneumonia would show as many recoveries.

There is one other reason for sending patients early to the hospital: that is the difference of time required for recovery.

*Report, 1840. †Earle. ‡Browne. || Report.

We have analyzed the reports of cures, in the Worcester hospital, and find the following results:

| Duration of insanity before entering hospital. | No. of cases. | Average length of disease before entering hospital. | Average time required for cure. |
|---|---|---|---|
| 1 month and under, | 115 | 18 days, - - - - - | 15½ weeks. |
| 1 to 3 months, - - | 145 | 71 days, - - - - - | 17 weeks. |
| 3 to 6 months, - - | 79 | 22 weeks, - - - - | 18 weeks. |
| 6 to 9 montns, - - | 32 | 8 months, - - - - | 23½ weeks. |
| 9 to 12 months, - | 36 | 11 months 3 weeks. | 29 weeks. |
| 1 to 5 years, - - - | 65 | 35 months 2 weeks. | 36 weeks. |
| 5 years and over, - | 17 | 122 months 3 weeks. | 39 weeks. |

Dr. Pritchard's analysis of the recoveries in the Gloucester asylum, gives 19 weeks as the time required to cure the cases of one month and less duration; and 33 weeks for the curing those of one to three month's standing.*

The time required for recovery increases, with the previous length of the disease, though not in exact ratio. Yet it is sufficiently manifest, that the delay of sending a lunatic to the asylum not only increases the expense of the cure, but also diminishes very rapidly the chance of the recovery.

Having now demonstrated how much is done for the cure of insanity, by the best institutions of other States, and how little is done for the same purpose in Kentucky, it is manifest, that our asylum falls very far short of the good success of theirs: and yet we offer our sincere opinion, that we may do as well as they. If we will put our asylum on as liberal and improved footing as others, if we will supply it as freely with medical and all other attendants, and means of occupation, if we will so frame our laws as to send every lunatic, in the very incipiency of the disease, to Lexington, we shall rescue many good citizens from the bondage of hopeless insanity, and save to the State a great part of the expense of maintaining them.

We now leave the whole matter in the hands of our enlightened Legislature; and while they remember, with satis-

*Pritchard, p. 103.

faction, that "Kentucky has the honor of being the first State in the Union," excepting Virginia, "to establish, at the expense and under the control of the State, an asylum for poor lunatics,"* and that long afterwards other States followed her noble example; they must also remember, that those States have made much more rapid improvements than she has, and outstripped her in this march of humanity. And as they took their first lessons of us, to establish their asylums, so we must now take our second lesson of them to raise our lunatic hospital up to the standard of the usefulness of theirs; and we earnestly beseech our philanthropic Legistors not to falter in this generous work, until insanity shall be shewn to be as curable at Lexington, as it is any where in the land. E. J.

*N. A. Review, xliv, 112.

Note.—In adddition to the authorities frequently referred to, in course of this article, we are indebted, for many of the foregoing facts, to the exceedingly valuable and interesting reports of the Prison Discipline Society, whose benevolent and indefatigable secretary, Rev. Louis Dwight, has searched into every lunatic asylum as well as every prison in our land. His reports embrace a vast variety of information relative to the various means and success of treating insanity in the different hospitals, and cannot be read without deep interest and profit, by the physician and philanthropist.

