# Erratum

**Published:** 1841-12

**Authors:** 


*Erratum.*—In page 470, second line from the bottom, for 1839–40, read 1838, '39 and '40.

